# Oligodendrocyte precursor cell–neuronal lysosomal pathway: A novel therapeutic target for neurodegenerative diseases

**DOI:** 10.4103/NRR.NRR-D-25-00625

**Published:** 2025-09-29

**Authors:** Li-Pao Fang, Yibo Zhao, Xianshu Bai

**Affiliations:** State Key Laboratory of Natural Medicines, Department of Pharmacology, School of Pharmacy, China Pharmaceutical University, Nanjing, Jiangsu Province, China; Pharmaceutical Research Center for Gender-specific Biology and Medicine, China Pharmaceutical University, Nanjing, Jiangsu Province, China; Department of Molecular Physiology, Center for Integrative Physiology and Molecular Medicine, University of Saarland, Homburg, Germany; Center for Gender-specific Biology and Medicine, University of Saarland, Homburg, Germany

**Figure 1:**
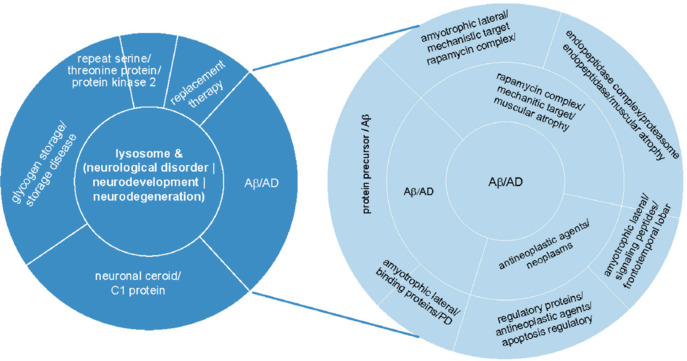
A comprehensive literature analysis reveals lysosomal dysfunction is linked to neurodegenerative and storage disorders. The analysis included 13,991 publications identified through a PubMed search using the term “lysosome” in combination with (“neurological disorder” OR “neurodevelopment” OR “neurodegeneration”) within titles and abstracts. The pie chart illustrates the distribution of research focus across different diseases based on keyword frequency. For example, focusing on AD, a subset of 3691 papers was analyzed, demonstrating a significant research interest in lysosomal mechanisms underlying AD pathology. Data analysis and graph generation were conducted using Python, and the final visualization was refined using Adobe InDesign 2025. Full details of analysis and source code are available on GitHub (Zhao et al., 2025). Aβ: Amyloid-beta; AD: Alzheimer’s disease.

Oligodendrocyte precursor cells (OPCs) tile the central nervous system ubiquitously, accounting for about 5% of the total cell population in the central nervous system. Beyond their role in myelination, OPCs actively shape neural circuits (Fang and Bai, 2023), by releasing neuromodulators, pruning synapses, maintaining the homeostasis of extracellular potassium concentration, and interacting with endothelial cells. These cells feature a small cell body with highly branched processes, enabling communication with neighboring cells. OPCs establish contacts at various sites of neurons, including synaptic connections with axons and direct physical interactions at the nodes of Ranvier and neuronal somata. Recently, Fang et al. (2025) identified a contact-dependent signaling mechanism between OPCs and neurons. This interaction was observed universally across brain gray matter, with 91%–99% of neurons contacted by OPC processes, a phenomenon also confirmed in human cortical neurons. Functional studies revealed that OPC-neuron contact promotes lysosomal exocytosis from neurons. Notably, when OPC processes were diminished, either by genetic ablation of L-type voltage-gated calcium channels in OPCs or acute OPC depletion via induced diphtheria toxin A expression, the frequency of neuron-OPC contacts decreased. Consequently, affected neurons exhibited aberrant accumulation of enlarged lysosomes and lipid droplet in the somata (indicating lysosomal dysfunction), as well as molecular hallmarks of neuronal senescence and neurodegeneration. Many studies have established that impaired lysosomal function and exocytosis are strongly associated with neurodegenerative diseases and cellular senescence (Samie and Xu, 2014; Stagi et al., 2014; Lee et al., 2022; Xie et al., 2022; **Figure 1**). Lysosomes function as the waste disposal and recycling system of a cell, degrading biomolecules, damaged organelles, and foreign particles such as bacteria or viruses. These degraded materials are expelled from the cell via lysosomal exocytosis, a process in which lysosomes traffic to the plasma membrane, fuse with it, and release their contents. This mechanism is critical for clearing neurotoxic aggregates, thereby protecting cells from damage and death. Thus, the control of neuronal lysosomal exocytosis and function by OPC may serve as a novel therapeutic target for neurodegenerative diseases.

Alzheimer’s disease (AD) exemplifies a condition where lysosomal dysfunction occurs in neurons. Impaired autolysosome acidification leads to the build-up of amyloid-beta (Aβ) selectively within enlarged autolysosomes, which occurs already about 4 months prior to the formation of extracellular plaques in the AD mouse model (Lee et al., 2022). Under normal circumstances, intracellular Aβ is largely secreted from the cells by lysosomal exocytosis. Inhibition of exocytosis causes a general increase of intracellular Aβ, both in and outside of lysosome, leading to synapse damage and memory impairment. In an early stage of AD pathology using Tg2576 (6 months) AD mouse models, cortical OPCs exhibited reduced arborization and subsequently fewer contacts on neuronal somata. Concomitantly, these neurons displayed pronounced lysosomal accumulation, suggesting that OPC morphological atrophy may act as an early contributor to AD pathology. Notably, at the late stage of AD pathology, the complexity of OPC morphology increases (Vanzulli et al., 2020). However, whether OPC-neuron contact is increased, or OPC-mediated neuronal lysosomal exocytosis is enhanced or not, is not clear. Intriguingly, while microglia near Aβ plaques show a disease-associated phenotype (Deczkowska et al., 2018), only OPCs, particularly those associated with Aβ plaques, display a senescence-like phenotype in both the brains of AD patients and mouse models (7.5 months APP/PS1 model) (Zhang et al., 2019). In addition, neuronal dendrites near OPCs show an accumulation of cargo-laden autolysosomes (Zhang et al., 2019), indicating that not only the OPC-neuron physical contact but also OPC function is critically important to neuronal lysosomal function and Aβ disposal in AD pathology. Strengthening OPC–neuron interactions or restoring OPC function in AD may represent promising therapeutic strategies, for example, transplanting “young” OPCs into the brain may represent a promising strategy to mitigate AD pathology. However, further experimental validation is required.

Parkinson’s disease serves as another compelling example of neurodegeneration involving lysosomal dysfunction. The disease is pathologically characterized by dopaminergic neuron loss in the substantia nigra. While the classical pathological hallmark involves accumulation of α-synuclein aggregates, emerging evidence indicates that impaired lysosomal degradation and exocytosis contribute significantly to this pathogenic cascade (Xie et al., 2022). A recent study has shown that OPCs in the mouse substantia nigra form tight contact with neuronal somata under physiological conditions (Fitzgerald et al., 2025). Although whether such contact is impaired in Parkinson’s disease condition is yet unknown, OPC-mediated neuronal lysosome exocytosis sheds light on a novel therapeutic strategy for Parkinson’s disease.

TMEM106B variants are associated with an increased risk of frontotemporal lobar degeneration. Overexpression of TMEM106B disrupts lysosomal trafficking, leading to enlarged lysosomes and their accumulation in neuronal somata (Stagi et al., 2014). Although the morphological changes in OPCs in frontotemporal lobar degeneration remain poorly understood, the observation that enlarged lysosomes exhibit reduced degradative capacity (de Araujo et al., 2020) suggests that increasing OPC-neuron contact could promote lysosomal exocytosis. Such enhancement might restore lysosomal activity, reduce intracellular neurotoxic protein levels, and potentially slow disease progression.

The majority of neurodegenerative diseases are associated with aging. In the aged brain, neurons undergo senescence, which is increasingly recognized as a key driver of cognitive decline and neurodegeneration. Notably, lysosomal function declines with age, leading to the aberrant accumulation of damaged proteins, organelles, and lipid droplets within cells, which further promotes neurodegeneration. Recent findings by Fang et al. (2025) reveal a novel mechanism underlying this lysosomal dysfunction: the loss of contact between neurons and OPCs may accelerate neuronal senescence in the aging brain. When OPC–neuron interactions are disrupted, neurons exhibit abnormal lysosomal accumulation and elevated p16INK4A expression, suggesting that OPCs play a direct role in modulating neuronal homeostasis through lysosomal pathways. However, in the aged mouse cortex (24–26 months old), while OPC arborization increases, OPC senescence also rises (Ogrodnik et al., 2021; Ma et al., 2024). This raises the possibility that although OPC-neuron contacts may increase in the aged brain, OPC-mediated lysosomal exocytosis in neurons could still be impaired. Taken together, these findings indicate that OPC-neuron contacts are essential for maintaining neuronal lysosomal function and metabolic health, but this regulatory mechanism depends on the presence of functionally intact OPCs.

Lysosomal storage disorders are inherited metabolic conditions caused by mutations in genes encoding lysosomal enzymes or associated proteins, leading to impaired degradation of cellular waste. The resulting accumulation of undegraded materials, such as glycosphingolipids and glycosaminoglycans, disrupts critical lysosomal functions, including autophagy, vesicle trafficking, and calcium homeostasis. Most lysosomal storage disorders stem from loss-of-function mutations that compromise enzyme folding, trafficking, or catalytic activity, ultimately driving progressive neurodegeneration (Samie and Xu, 2014). OPCs support neuronal health via contact-dependent modulation of lysosomal exocytosis. While the role of OPCs in lysosomal storage disorders remains poorly understood, one potential therapeutic strategy could involve enhancing OPC-neuron interactions. By restoring these interactions, it may be possible to improve neuronal lysosomal clearance, reduce metabolic stress, and mitigate disease progression.

The contact-dependent regulation of neuronal metabolism and function by OPCs highlights a novel therapeutic strategy for neurodegenerative diseases, particularly through modulation of lysosomal function. Lysosomes serve not only as the waste disposal system of the cell but also as critical signaling hubs. Lysosomal exocytosis facilitates the clearance of neurotoxic aggregates while simultaneously enabling intercellular communication. Glial cells have been shown to release adenosine triphosphate via lysosomal exocytosis. Adenosine triphosphate and its metabolic derivatives (e.g., adenosine) play key roles in modulating neural networks. Given the spatial specificity of lysosomal exocytosis at OPC-neuron contact sites, we hypothesize that adenosine triphosphate (or its hydrolyzed products) could act on purinergic receptors in OPCs, thereby mediating bidirectional neuron-OPC signaling. However, there are still open fundamental questions: How is physical contact between neurons and OPCs initiated? What molecular cascades are triggered by lysosomally released contents at these junctions? Answering these questions will open multi-target therapeutic strategies for neurodegenerative diseases, combining lysosomal restoration, enhancement of OPC-neuron interaction and intervention in signaling cascades between these cells.

*We thank Professor Frank Kirchhoff (Molecular Physiology, University of Saarland, Germany) for his generous support to our work.*

*This work was supported by Deutsche Forschungsgemeinschaft (BA 8014/1-1 to XB),* *University of Saarland (NanoBioMed Young Investigator* *grant 2021 to XB, Anschubsfinanzierung2024 to XB, HOMFORExzellenz2025 and Anschubsfinanzierung2025 to LPF) and the China Pharmaceutical University (Undergraduate Internship Program to YZ).*

**Additional file:** *Open peer review report 1.*

OPEN PEER REVIEW REPORT 1
