# Supplementary material for: Oligodendrocyte precursor cell–neuronal lysosomal pathway: A novel therapeutic target for neurodegenerative diseases
Source: Neural Regen Res. 2025 Sep 29;21(6):2355–6. doi: 10.4103/NRR.NRR-D-25-00625 (PMC13211787; doi:10.4103/NRR.NRR-D-25-00625)
Supplement: Supplementary file 1 [file NRR-21-2355_Suppl1.pdf]

OPEN PEER REVIEW REPORT 1

Name of journal: Neural Regeneration Research
Manuscript NO: NRR-D-25-00625
Title: Oligodendrocyte precursor cell-neuronal lysosomal pathway: A novel therapeutic target for neurodegenerative diseases
Reviewer's Name: Juliana Helena Castro e Silva
Reviewer's country: Italy

COMMENTS TO AUTHORS

This is a perspective article in which the authors highlight recent evidence on the role of oligodendrocyte precursor cell communication with neurons, with a focus on lysosomal dysfunction and transfer. To discuss the topic, the author uses recent, updated, and impactful references that demonstrate that OPC contact to neurons is essential to prevent lysosomal accumulation and that a loss in this interaction might have a role in neuronal lysosomal function, including autophagy and vesicle trafficking, leading to neurodegeneration.

The article is well written overall, cohesive, and concise. The authors properly shed light on what is missing for the subject and asked important questions. However, I would suggest alterations to better adequately address the conclusions taken by the authors:

1) Briefly discuss that OLs can support neuronal function and vice-versa by other means, including lysosomal signaling.

2) L49 "Intriguingly, OPCs, but not the other glial cell types, especially those associated with Aβ plaques, exhibit a senescence-like phenotype in both the brains of AD patient and mouse model (7.5 months APP/PS1 model) (Zhang et al., 2019)." Although this might be correct for the present reference, other pieces of evidence have demonstrat-ed, for example, that also microglia assume very specific gene expression signatures in neurodegeneration (For consultation: https://doi.org/10.3389/fncel.2024.1476461;https://doi.org/10.1016/j.cell.2018.05.003) Overall, this is a compelling and innovative perspective that adds valuable insight to the field.
